# Supplementary material for: Enhanced Sensitivity and Accuracy of Tb3+-Functionalized Zirconium-Based Bimetallic MOF for Visual Detection of Malachite Green in Fish
Source: Foods. 2024 Sep 9;13(17):2855. doi: 10.3390/foods13172855 (PMC11395321; doi:10.3390/foods13172855)
Supplement: Supplementary file 1 [file foods-13-02855-s001.zip › foods-3172147-supplementary.pdf]

Supporting Material

Enhanced Sensitivity and Accuracy of Tb^{3+}-Functionalized Zirconium-Based Bimetallic MOF for Visual Detection of Malachite Green in Fish

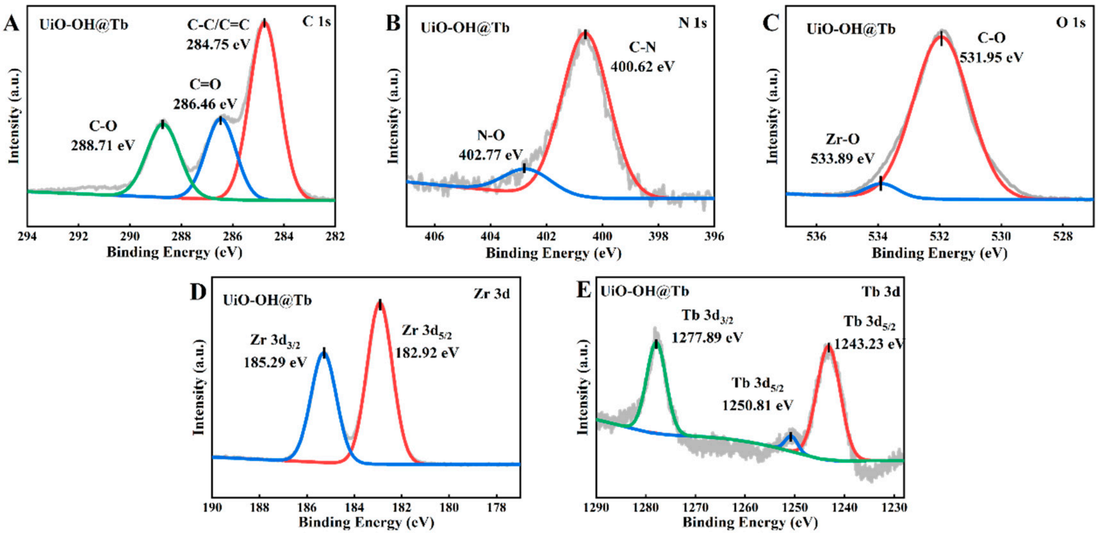

Figure S1. XPS spectra of UiO-OH@Tb. High resolution C 1s (A), N 1s (B), O 1s (C), Zr 3d (D), Tb 3d (E) for UiO-OH@Tb.

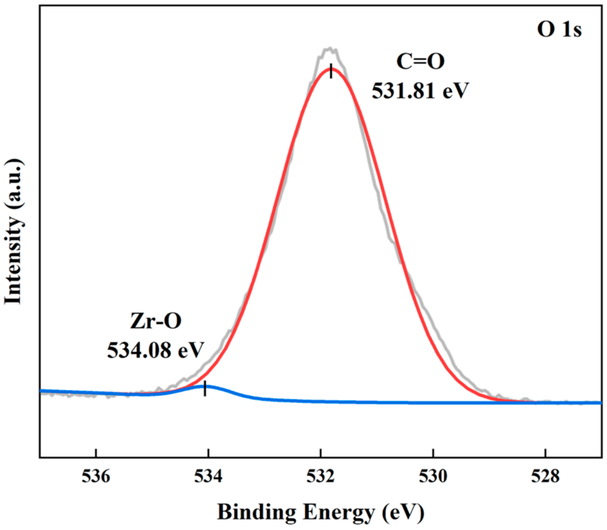

Figure S2. XPS spectra of UiO-OH. High resolution O 1s for UiO-OH.

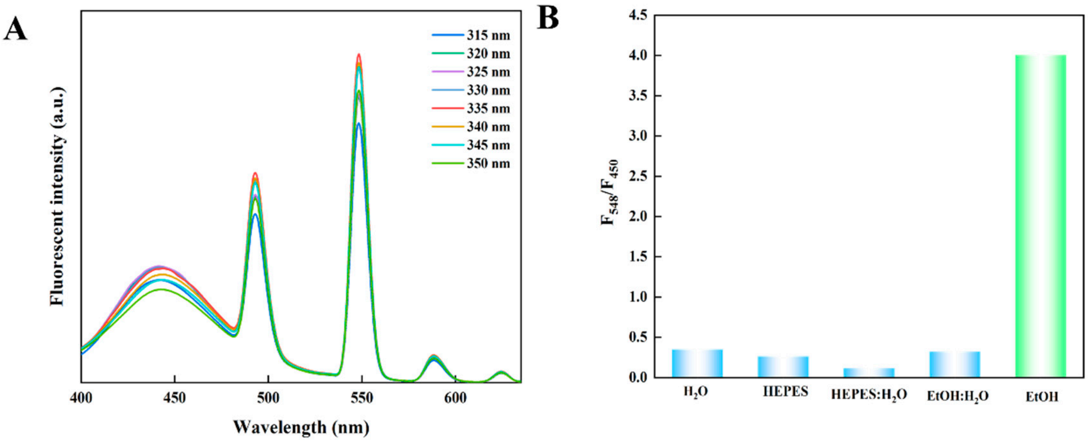

Figure S3. (A) Fluorescence spectra of UiO-OH@Tb under different excitations. (B) Effect of different buffer solvents on the fluorescence ratio (F_{548}/F_{450}) of UiO-OH@Tb.

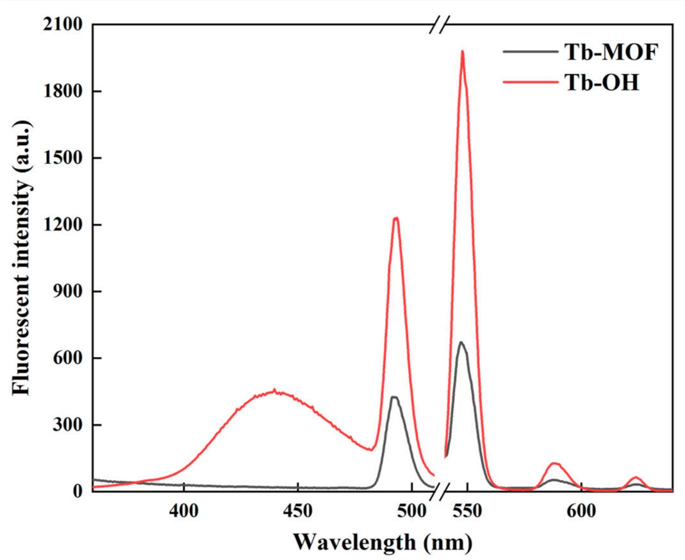

Figure S4. Fluorescence spectra of Tb-MOF, Tb-OH.

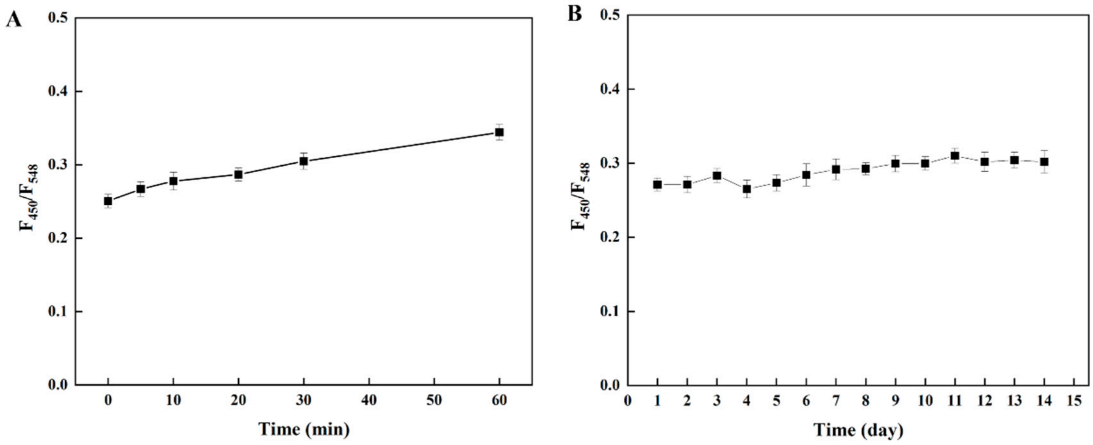

Figure S5. (A) Effect of UV lamp irradiation time on the fluorescence ratio of UiO-OH@Tb (F_{450}/F_{548}). (B) Effect of storage time on the fluorescence ratio of UiO-OH@Tb (F_{450}/F_{548}).

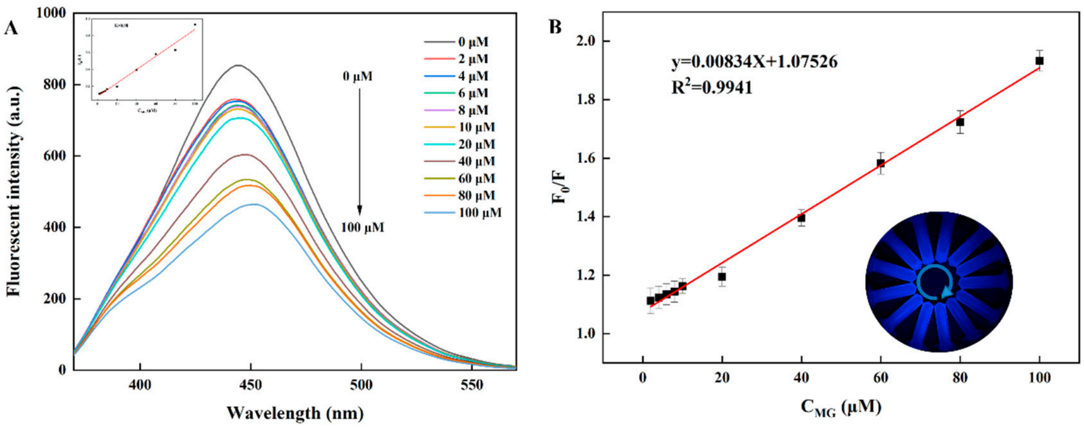

Figure S6. (A) Fluorescence emission spectra of UiO-OH after adding different concentrations of MG (0–100 μM), Inset: Stern-Volmer curve of UiO-OH after MG treated. (B) The linear relationship between F_0/F and the concentration of MG (0–100 μM).

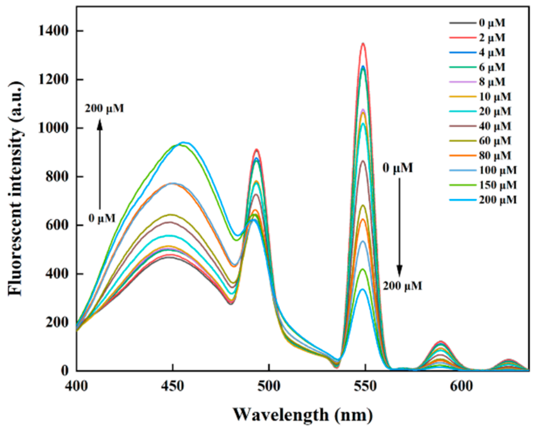

Figure S7. Fluorescence emission spectra of UiO-OH@Tb after adding different concentrations of MG (0–200 μM).

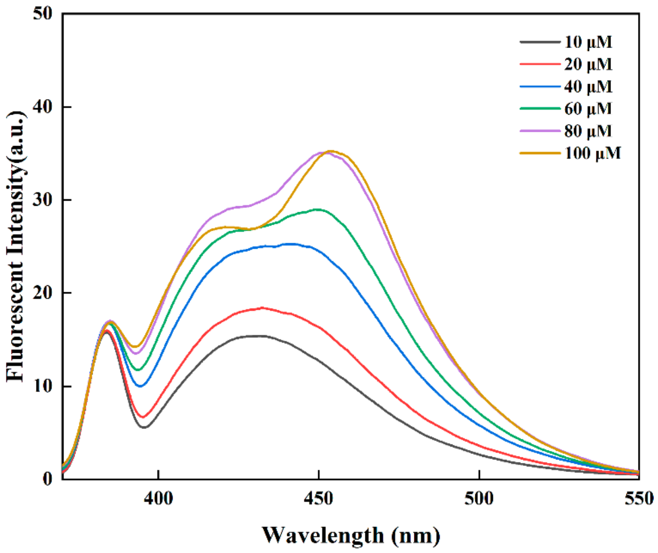

Figure S8. Fluorescence spectra of different concentrations (10-100 μM) of MG.

Table S1. Quantum yield of Tb-MOF, Tb-OH.

	A	I	η	QY
Tb-MOF	0.044	13.458	1.33	0.36%
Tb-OH	0.047	699.955	1.33	16.99 %
QS	0.046	2176.769	1.33	54%

The calculation formula is as follows [1]

$$\mathbf{QY=QS\times(I_U/Ist)\times(Ast/A_U)\times(\eta_u^2/\eta_{st}^2)}$$

In the formula: QY is the quantum yield, I is the fluorescence front area, A is the absorbance, and η is the refractive index. The subscript "u" represents the sample with unknown quantum yield, "st" represents the quinine sulfate standard, and the subscript "u" represents the sample with unknown quantum yield. QS is quinine sulfate.

References

[1] S.Y. Tang, D. Chen, X.M. Li, C.X. Wang, T.T. Li, J.X. Ma, G.Q. Guo, Q.L. Guo, Promising energy transfer system between fuorine and nitrogen Co-doped graphene quantum dots and Rhodamine B for ratiometric and visual detection of doxycycline in food, *Food Chemistry*, 388 (2022).
